# Investigation of the association between cardio-metabolic risk factors, neurotrophins and gastric hormones among apparently healthy women: A cross-sectional analysis

**DOI:** 10.34172/jcvtr.2022.10

**Published:** 2022-03-06

**Authors:** Reihaneh Zeinalian, Dorsa Arman Moghadam, Naseh Pahlavani, Neda Roshanravan, Mohammad Alizadeh, Masoumeh Jabbari, Sorayya Kheirouri

**Affiliations:** ^1^Department of Nutrition in Community, Faculty of Nutrition, Tabriz University of Medical Sciences, Tabriz, Iran; ^2^Department of Nutrition, Science and Research Branch, Islamic Azad University, Tehran, Iran; ^3^Department of Nutrition, School of Medicine, Hamadan University of Medical Sciences, Hamadan, Iran; ^4^Department of Clinical Biochemistry and Nutrition, Faculty of Medicine, Kurdistan University of Medical Sciences, Sanandaj, Iran; ^5^Cardiovascular Research Center, Tabriz University of Medical Sciences, Tabriz, Iran; ^6^Nutrition Research Center, Tabriz University of Medical Sciences, Tabriz, Iran; ^7^Faculty of Nutrition Sciences and Food Industry, Department of Community Nutrition, Shahid Beheshti University of Medical Sciences, Tehran, Iran; ^8^Department of Nutrition, Faculty of Nutrition, Tabriz University of Medical Sciences, Tabriz, Iran

**Keywords:** Cardio-metabolic, NGF, BDNF, Ghrelin, Obestatin

## Abstract

***Introduction:*** Although, some evidence has shown that obestatin, ghrelin, and neurotrophic factors can be involved in the development of cardio-metabolic risk factors, there are some contradictions in this regard. We aimed to investigate the association of serum neurotrophic factors and some gastric hormones with cardio-metabolic risk factors among apparently healthy women.

***Methods:*** In the present study, 90 apparently healthy women were recruited by a convenient sampling method from the nutrition counseling clinic in Tabriz, Iran. All participants need dietary counseling for weight loss and were recruited before receiving any dietary interventions. Anthropometric, biochemical, physical activity, and blood pressure (BP) measurements, as well as dietary assessment, were done in all participants.

***Results:*** Women who were in the highest tertile of serum obestatin levels (OR=0.118, 95% CI:0.035-0.396) had a significantly lower risk for development of hypertriglyceridemia compared to the reference group (P_trend_ < 0.001). Participants in the highest tertile of serum ghrelin had a significant lower risk of hypertriglyceridemia, hyperglycemia, low HDL-C, and MetS (P_trend_ < 0.05). Women who were in the higher tertile of serum NGF levels had a significantly lower risk for development of hypertriglyceridemia after adjustment for potential confounding variables (OR=0.091, 95% CI: 0.023-0.361 and OR=0.193, 95% CI: 0.057-0.649 respectively).

***Conclusion:*** In the current study serum levels of obestatin, NGF, and ghrelin were associated with some cardio-metabolic risk factors such as hypertriglyceridemia and MetS. It seems that these factors are associated with metabolic regulation. However, further studies are needed to substantiate this claim.

## Introduction

The role of metabolic dysregulation in the pathogenesis of various chronic diseases including cardiovascular diseases, diabetes, cancer, and other chronic conditions, has recently become a challenging topic.^1^ Cardio-metabolic risk (CMR), as combined vascular and metabolic components, refers to various risk factors such as hypertension, dyslipidemia, and abdominal obesity that increase the probability of experiencing cardiovascular events.^2^ Cardio-metabolic dysregulations because of chronic sub-clinical inflammation and oxidative stress, leads to the adipokines dysregulation. Adipokines are the signaling proteins with some key roles in metabolic regulation.^3^

Neurotrophins including nerve growth factor (NGF) family and brain derived neurotrophic factor (BDNF) are small polypeptides with potent effects on health and well-being of the neuronal and non-neuronal cells.^4,5^ Recently, NGF and BDNF as adipokines are taken into consideration due to their possible roles in some metabolic disorders such as diabetes mellitus^6,7^, atherosclerosis^8^ and metabolic syndrome (MetS).^9^ Despite the contradictory results^10^, the growing body of evidence suggests that low levels of circulating BDNF and NGF were significantly associated with high body mass index (BMI), hemoglobin A1C (HbA1C) and hypertriglyceridemia.^11^

The circulating levels of obestatin, ghrelin and obestatin /ghrelin ratio have recently been a subject of intense research in cardio-metabolic disease.^12^ Obestatin, a peptide hormone released from the stomach, is encoded by the pre-proghrelin gene.^13,14^ Ghrelin is produced predominantly by the gastrointestinal tract and involved in the regulation of energy balance.^11,15^ This peptide acts as an insulin secretagogue, however the exact mechanism of its action on metabolism regulation is less clear.^16^

Although, some evidence has shown that obestatin, ghrelin and neurotrophic factors can be involved in development of CMR by affecting specific biological processes; there are some contradictions in this regard. Previous studies have often measured the serum/plasma level of BDNF, NGF, obestatin and ghrelin in MetS, obese and/or healthy groups and compared these levels between groups. There are so limited risk assessment studies in this regard; specially, regression risk assessment on the mentioned adipokines and neurotrophins and CMR factors. So, in the present study we conducted the risk assessment analysis of BDNF, NGF, obestatin and ghrelin with CMR factors among apparently healthy women.

## Materials and methods

### Study participants

In the present study, ninety apparently healthy women were recruited by a convenient sampling method from the Asad Abadi nutrition counseling clinic in Tabriz, Iran from January to June 2015. All participants need dietary counseling for weight loss and were recruited before receiving any dietary interventions. The Ethical Committee of Tabriz University of Medical Sciences, Tabriz, Iran approved the study protocol, and all participants have signed the written informed consent form before the study beginning. (Ethical code: TBZMED.REC.1394.111). For this cross-sectional analysis, inclusion criteria include pre-menopause women aged between 30-50 years. Exclusion criteria include: medical history of any chronic, endocrine, infectious, inflammatory, or psychiatric diseases, receiving medical and nutritional/dietary therapy (i.e., weight loss, anti-inflammatory, anti-hypertensive, corticosteroid and estrogen drugs, contraceptives and therapeutic supplements) during the three months before the study, BMI ≥ 40 kg/m^2^, alcohol consumption, smoking, being pregnant or breast-feeding. The American Heart Association (AHA) criteria^17^ were used to assess MetS status among the study participants. Based on this criteria, women who have three or more the following risk factors were classified into MetS group: waist circumference (WC) > 88 cm, fasting blood sugar (FBS) ≥ 100 mg/dL, high-density lipoprotein *cholesterol* (HDL-C) < 50 mg/dL, triglyceride (TG) ≥ 150 mg/dL, blood pressure (BP) ≥ 130/85 mm Hg.

### Dietary and anthropometric measurements

A 3-day dietary record questionnaire was used for the dietary assessment of all participants. A trained nutritionist explained to all participants the instruction on how to fill the questionnaire. The pre-structured dietary record including separate pages for recording full details of all consumed meals and snacks, was given to them for all three days. Participants were asked to fill the dietary questionnaire for two usual and one holiday in a week. All records were checked after completion, and if needed, the trained nutritionist requested the participants for more clarification by phone call. We used Nutritionist-4 software for analysis of dietary records for extracting nutrients and calorie intake. A digital scale measured bodyweight while subjects wore a light gown and barefoot to the nearest 0.10 kilogram. A wall-mounted stadiometer was used to measure barefooted height to the nearest 0.5 cm. BMI was calculated based on the following formula: weight (kg)/ height(m^2^). A tape meter was used to WC measurement nearest 0.5 cm at the midpoint between the lower costal margin and iliac crest. Also, hip circumference was measured around the widest portion of the buttocks. For calculation of Waist-to-Hip Ratio (WHR), waist measurement (cm) was divided by hip measurement (cm).

### Physical activity and BP

After 5-minute rest in a sitting position, BP measurement was done twice, with a 15-minute interval, by a calibrated sphygmomanometer. The final values of BP for all participants were estimated as the mean of two readings. The short form of the International Physical Activity Questionnaire (IPAQ) was used to determine the physical activity level of participants (validated among Iranian adults)^18^, and reported as a standard Metabolic Energy Turnover into MET-minutes/week. Then, physical activity levels among participants were classified in the following three categories: 1) low ( < 600 MET-minutes/week), 2) moderate (between 600 to 2999 MET-minutes/week), and 3) high ( ≥ 3000 MET-minutes/week).

### Biochemical assessments

Blood samples of all participants were collected after overnight fasting. All samples were centrifuged at 2000 rpm for 10 minutes at 4° C to separate serum samples. All aliquots were stored at -70°C until biochemical analysis. Total serum cholesterol, HDL-C, and TG were determined by enzymatic colorimetric methods with a commercially available kit (Pars Azmone, Tehran, Iran) on an automatic analyzer (Abbott, model Alcyon 300, USA). By the glucose oxidase method, FBS was determined. Serum ghrelin, obestatin, NGF, and BDNF levels were measured using commercially available ELISA kits (Hangzhou Eastbiopharm CO, LTD).

### Statistical analysis

Continuous variables were presented as mean ± standard deviation (SD) and proportional data as frequency (percent). Binary logistic regression analysis was used to assess the association between CMR factors (including hypertriglyceridemia, hyperglycemia, low HDL-C, central obesity, hypertension (HTN), and MetS with serum obestatin, ghrelin, BDNF and, NGF in crude and adjusted models. In the adjusted model, analyses were adjusted for BMI, age, physical activity level, and dietary energy intake. All analyses were done by SPSS 22 Software (IBMSPSS statistics, Chicago, IL). *P* values less than 0.05 were considered statistically significant.

## Results

### General characteristics of the study population

In this cross-sectional analysis, ninety apparently healthy women (mean age of 37.30 ± 6.24 years) were included. General characteristics of the participants including anthropometric, dietary intake, and metabolic measurements were reported in Table 1. Based on the AHA criteria, among these participants, twenty women had high BP (Systolic BP/Diastolic BP ≥ 130/85 mm Hg), forty-six were hyperglycemic (FBS ≥ 100 mg/dL), thirty-two had hypertriglyceridemia (serum TG ≥ 150 mg/dL), fifty-four had low HDL-C ( < 50 mg/dL), and eighty-nine had central obesity (WC > 88 cm). Also, forty-seven women had MetS diagnostic criteria. The mean ± SD of serum obestatin, NGF, BDNF and ghrelin among the participants were 4.08 ± 0.51 ng/mL, 312.64 ± 59.71 pg/mL, 7.42 ± 1.54 ng/mL and 2.13 ± 0.51 ng/mL, respectively (Table 1).

**Table 1:** General characteristics of the study population

**Variables**		**Values**
Age (year) ^a^		37.30 ± 6.24
Waist circumference (cm) ^a^		108.07 ± 10.54
Hip circumference (cm) ^a^		114.00 ± 8.61
Waist to hip ratio ^a^		0.94 ± 0.06
Body mass index (BMI) (kg/m^2^)		33.52 ± 3.72
Systolic blood pressure (mm Hg) ^a^		114.27 ± 19.75
Diastolic blood pressure (mm Hg) ^a^		75.73 ± 11.71
Fasting blood sugar (FBS) (mg/dL) ^a^		100.47 ± 9.82
Triglyceride (TG) (mg/dL) ^a^		130.31 ± 59.76
High density lipoprotein cholesterol (HDL-C) (mg/dL) ^a^		46.18 ± 9.31
Dietary energy intake (kcal) ^a^		2006.25 ± 510.58
Dietary Protein intake (gr) ^a^		65.84 ± 19.22
Dietary Fat intake (gr) ^a^		74.29 ± 21.79
Dietary carbohydrate intake (gr) ^a^		289.02 ± 82.19
Serum Obestatin (ng/mL) ^a^		4.08 ± 0.51
Serum NGF (pg/mL) ^a^		312.64 ± 59.71
Serum BDNF (ng/mL) ^a^		7.42 ± 1.54
Serum Ghrelin (ng/mL) ^a^		2.13 ± 0.51
Physical activity level (METs) ^b^	Low	35 (38.9)
	Moderate	55 (61.1)
Hypertension (HTN) ( ≥ 130/85 mm Hg) ^b^	No	70 (77.8)
	Yes	20 (22.2)
Hyperglycemia ( ≥ 100 mg/dL) ^b^	No	44 (48.9)
	Yes	46 (51.1)
Hypertriglyceridemia ( ≥ 150 mg/dL) ^b^	No	58 (64.4)
	Yes	32 (35.6)
Low HDL-C ( < 50 mg/dL)^b^	No	36 (40)
	Yes	54 (60)
High waist circumference ( > 88 cm) ^b^	No	1 (1.1)
	Yes	89 (98.9)
Metabolic Syndrome (MetS) ^b^	No	43 (47.8)
	Yes	47 (52.2)

Abbreviations: NGF, nerve growth factor; BDNF, brain derived neurotrophic factor ^a^Data were reported based on mean ± SD ^b^Data were reported based on frequency(percent)

### CMR factors and serum obestatin

The association of CMR factors and serum obestatin tertiles in the study population was shown in Table 2. Women who were in the second (OR = 0.262, 95% CI: 0.089-0.773) and third (OR = 0.118, 95% CI: 0.035-0.396) tertiles of serum obestatin had significantly lower risk of hypertriglyceridemia compared to the reference group (P _trend_ < 0.001). This association remained significant after adjustment for potential confounding variables, including BMI, age, physical activity level and dietary energy intake. Risk of MetS was significantly lower among the women only in the second tertile of serum obestatin compared to the reference group (OR = 0.261, 95% CI: 0.088-0.769). This association remained significant after adjustment for BMI, age, physical activity level and energy intake (OR = 0.268, 95% CI: 0.083-0.865, P _trend_ = 0.226). There was no significant association between the risk of HTN, central obesity, low HDL-C, and hyperglycemia among serum obestatin tertiles (P _trend_ > 0.05).

**Table 2:** The association of cardio-metabolic risk factors with serum Obestatin tertiles in the study population

**Variables**		**Tertiles of obestatin**			
		**T1** **(≤3.9 ng/mL)**	**T2** **(3.9-4.3 ng/mL)**	**T3** **(> 4.3 ng/mL)**	***P*** ** for trend**
Hypertriglyceridemia ( ≥ 150 mg/dL)	Normal TG (%)/ High TG (%)	11 (37.9)/ 18 (62.1)	21 (70)/ 9 (30)	26 (83.9)/ 5 (16.1)	< 0.001^*^
	Model 1 OR (95% CI)	Ref.	0.262 (0.089-0.773)^*^	0.118 (0.035-0.396)^*^	
	Model 2 OR (95% CI)	Ref.	0.244 (0.075-0.801)^*^	0.099 (0.027-0.371)^*^	
Hyperglycemia ( ≥ 100 mg/dL)	Normal FBS(%)/ High FBS (%)	13 (44.8)/ 16 (55.2)	16 (53.3)/ 14 (46.7)	15 (48.4)/ 16 (51.6)	0.852
	Model 1 OR (95% CI)	Ref.	0.711 (0.255-1.98)	0.867 (0.314-2.39)	
	Model 2 OR (95% CI)	Ref.	0.775 (0.262-2.28)	0.0889 (0.304-2.59)	
Low HDL-C ( < 50 mg/dL)	Normal HDL(%)/ low HDL (%)	10 (34.5)/ 19 (65.5)	15 (50)/ 15 (50)	11 (35.5)/ 20 (64.5)	0.829
	Model 1 OR (95% CI)	Ref.	0.526 (0.185-1.50)	0.957 (0.331-2.76)	
	Model 2 OR (95% CI)	Ref.	0.505 (0.162-1.57)	1.22 (0.389-3.82)	
Central obesity (waist circumference > 88 cm)	Normal (%)/Obese (%)	0 (0)/ 29 (100)	0 (0)/ 30 (100)	1 (3.2)/ 30 (96.8)	0.997
	Model 1 OR (95% CI)	Ref.	-	-	
	Model 2 OR (95% CI)	Ref.	-	-	
HTN ( ≥ 130/85 mm Hg)	Normal (%)/ HTN (%)	21 (72.4)/ 8 (27.6)	25 (83.3)/ 5 (16.7)	24 (77.4)/ 7 (22.6)	0.628
	Model 1 OR (95% CI)	Ref.	0.525 (0.149-1.84)	0.766 (0.237-2.47)	
	Model 2 OR (95% CI)	Ref.	0.615 (0.154-2.46)	0.750 (0.209-2.69)	
MetS	Healthy (%)/MetS (%)	9 (31)/ 20 (69)	19 (63.3)/ 11 (36.7)	15 (48.4)/ 16 (51.6)	0.226
	Model 1 OR (95% CI)	Ref.	0.261 (0.088-0.769)^*^	0.480 (0.167-1.38)	
	Model 2 OR (95% CI)	Ref.	0.268 (0.083-0.865)^*^	0.536 (0.173-1.66)	

Abbreviations: HTN, hypertension; HDL-C, high density lipoprotein cholesterol; MetS, metabolic syndrome; BMI, body mass index Data were reported based on the binary logistic regression analysis. Model 1: Crude model Model 2: Adjusted for BMI, Age, physical activity level and energy intake *P < 0.05 is significant.

### CMR factors and serum ghrelin

In Table 3, the association of CMR factors and serum ghrelintertiles in the study population was shown. Participants in the highest tertile of serum ghrelin had a significant lower risk of hypertriglyceridemia (P _trend = _0.003), hyperglycemia (P _trend = _0.021), Low HDL-C (P _trend = _0.006), and MetS (P _trend_ < 0.001) in both crude and adjusted models. We found no significant associations between the risk of central obesity and HTN among serum ghrelin tertiles in both crude and adjusted models (P _trend_ > 0.05).

**Table 3:** The association of cardio-metabolic risk factors with serum Ghrelin tertiles in the study population

**Variables**		**Tertiles of Ghrelin**			
		**T1** **(≤1.98 ng/mL)**	**T2** **(1.98-2.34 ng/mL)**	**T3** **(> 2.34 ng/mL)**	***P*** ** for trend**
Hypertriglyceridemia ( ≥ 150 mg/dL)	Normal TG (%)/ High TG (%)	13 (48.1)/14 (51.9)	15 (55.6)/ 12 (44.4)	30 (83.3)/ 6 (16.7)	0.003^*^
	Model 1 OR (95% CI)	Ref.	0.743 (0.255-2.16)	0.186 (0.058-0.591)^*^	
	Model 2 OR (95% CI)	Ref.	0.603 (0.195-1.86)	0.161 (0.048-0.542)^*^	
Hyperglycemia ( ≥ 100 mg/dL)	Normal FBS(%)/ High FBS (%)	9 (33.3)/ 18 (66.7)	13 (48.1)/ 14 (51.9)	22 (61.1)/ 14 (38.9)	0.021^*^
	Model 1 OR (95% CI)	Ref.	0.538 (0.179-1.61)	0.318 (0.112-0.904)^*^	
	Model 2 OR (95% CI)	Ref.	0.429 (0.132-1.39)	0.262 (0.085-0.813)^*^	
Low HDL-C ( < 50 mg/dL)	Normal HDL(%)/ low HDL (%)	8 (29.6)/ 19 (70.4)	7 (25.9)/ 20 (74.1)	21 (58.3)/ 15 (41.7)	0.006^*^
	Model 1 OR (95% CI)	Ref.	1.20 (0.365-3.96)	0.301 (0.104-0.867)^*^	
	Model 2 OR (95% CI)	Ref.	0.905 (0.247-3.32)	0.201 (0.060-0.674)^*^	
Central obesity (waist circumference > 88 cm)	Normal (%)/Obese (%)	0 (0)/ 27 (100)	1 (3.7)/ 26 (96.3)	0 (0)/ 36 (100)	0.997
	Model 1 OR (95% CI)	Ref.	-	-	
	Model 2 OR (95% CI)	Ref.	-	-	
HTN ( ≥ 130/85 mm Hg)	Normal (%)/ HTN (%)	19 (70.4)/ 8 (29.6)	22 (81.5)/ 5 (18.5)	29 (80.6)/ 7 (19.4)	0.465
	Model 1 OR (95% CI)	Ref.	0.540 (0.151-1.93)	0.573 (0.178-1.84)	
	Model 2 OR (95% CI)	Ref.	0.403 (0.100-1.62)	0.608 (0.170-2.17)	
MetS	Healthy (%)/MetS (%)	7 (25.9)/ 20 (74.1)	10 (37)/ 17 (63)	26 (72.2)/ 10 (27.8)	< 0.001^*^
	Model 1 OR (95% CI)	Ref.	0.595 (0.186-1.90)	0.135 (0.044-0.416)^*^	
	Model 2 OR (95% CI)	Ref.	0.374 (0.100-1.39)	0.086 (0.023-0.320)^*^	

Abbreviations: HTN, hypertension; HDL-C, high density lipoprotein cholesterol; MetS, metabolic Syndrome; BMI, body mass index Data were reported based on the binary logistic regression analysis. Model 1: Crude model Model 2: Adjusted for BMI, Age, physical activity level and energy intake *P < 0.05 is significant.

### CMR factors and serum NGF

The association of CMR factors and serum NGF tertiles in the study population was shown in Table 4. Women who were in the higher tertile of serum NGF levels had significantly lower risk of hypertriglyceridemia (OR = 0.103, 95% CI: 0.028-0.374, P _trend_ = 0.001) and MetS (OR = 0.220, 95% CI: 0.074-0.648, P _trend_ = 0.008) compared to the reference group. These associations remained significant for hypertriglyceridemia and MetS risk after adjustment for potential confounding variables including BMI, age, physical activity level and dietary energy intake (OR = 0.091, 95% CI: 0.023-0.361 and OR = 0.193, 95% CI: 0.057-0.649 respectively). Risk of hyperglycemia was significantly lower among the participants only in the second tertile of serum NGF compared to the reference group in both crude and adjusted models (OR = 0.278, 95% CI: 0.091-0.845 vs. OR = 0.240, 95% CI: 0.074-0.779, P _trend_ = 0.130). There were no significant associations between the risk of HTN, central obesity, and low HDL-C with serum NGF tertiles in both crude and adjusted models (P _trend_ > 0.05).

**Table 4:** The association of cardio-metabolic risk factors with serum NGF tertiles in the study population

**Variables**		**Tertiles of NGF**			
		**T1** **(≤290.19 pg/mL)**	**T2** **(290.19-333.18 pg/mL)**	**T3** **(> 333.18 pg/mL)**	***P*** ** for trend**
Hypertriglyceridemia ( ≥ 150 mg/dL)	Normal TG (%)/ High TG (%)	12 (44.4)/ 15 (55.6)	15 (53.6)/ 13 (46.4)	31 (88.6)/ 4 (11.4)	0.001^*^
	Model 1 OR (95% CI)	Ref.	0.693 (0.240-2.00)	0.103 (0.028-0.374)^*^	
	Model 2 OR (95% CI)	Ref.	0.553 (0.179-1.70)	0.091 (0.023-0.361)^*^	
Hyperglycemia ( ≥ 100 mg/dL)	Normal FBS(%)/ High FBS (%)	9 (33.3)/ 18 (66.7)	18 (64.3)/ 10 (35.7)	17 (48.6)/ 18 (51.4)	0.130
	Model 1 OR (95% CI)	Ref.	0.278 (0.091-0.845)^*^	0.529 (0.187-1.49)	
	Model 2 OR (95% CI)	Ref.	0.240 (0.074-0.779)^*^	0.417 (0.134-1.30)	
Low HDL-C ( < 50 mg/dL)	Normal HDL(%)/ low HDL (%)	10 (37)/ 17 (63)	10 (35.7)/ 18 (64.3)	16 (45.7)/ 19 (54.3)	0.449
	Model 1 OR (95% CI)	Ref.	1.05 (0.353-3.17)	0.699 (0.250-1.94)	
	Model 2 OR (95% CI)	Ref.	0.841 (0.263-2.68)	0.645 (0.207-2.00)	
Central obesity (waist circumference > 88 cm)	Normal (%)/Obese (%)	0 (0)/ 27 (100)	0 (0)/ 28 (100)	1 (2.9)/ 34 (97.1)	0.995
	Model 1 OR (95% CI)	Ref.	-	-	
	Model 2 OR (95% CI)	Ref.	-	-	
HTN ( ≥ 130/85 mm Hg)	Normal (%)/ HTN (%)	19 (70.4)/ 8 (29.6)	21 (75)/ 7 (25)	30 (85.7)/ 5 (14.3)	0.387
	Model 1 OR (95% CI)	Ref.	0.792 (0.241-2.60)	0.396 (0.113-1.39)	
	Model 2 OR (95% CI)	Ref.	0.794 (0.215-2.93)	0.539 (0.135-2.15)	
MetS	Healthy (%)/MetS (%)	8 (29.6)/ 19 (70.4)	12 (42.9)/ 16 (57.1)	23 (65.7)/ 12 (34.3)	0.008^*^
	Model 1 OR (95% CI)	Ref.	0.561 (0.184-1.71)	0.220 (0.074-0.648)^*^	
	Model 2 OR (95% CI)	Ref.	0.421 (0.125-1.41)	0.193 (0.057-0.649)^*^	

Abbreviations: HTN, hypertension; HDL-C, high density lipoprotein cholesterol; MetS, metabolic syndrome; NGF, nerve growth factor, BMI, body mass index Data were reported based on the binary logistic regression analysis. Model 1: Crude model Model 2: Adjusted for BMI, Age, physical activity level and energy intake *P < 0.05 is significant.

### CMR factors and serum BDNF

The association of CMR factors and serum BDNF tertiles in the study population was shown in Table 5. There was no significant association between serum BDNF tertiles and risk of hypertriglyceridemia, hyperglycemia, central obesity, low HDL-C, HTN, and MetS in both crude and adjusted models (P _trend_ > 0.05).

**Table 5:** The association of cardio-metabolic risk factors with serum BDNF tertiles in the study population

**Variables**		**Tertiles of BDNF**			
		**T1** **(≤6.69 ng/mL)**	**T2** **(6.69-8.03 ng/mL)**	**T3** **(> 8.03 ng/mL)**	***P*** ** for trend**
Hypertriglyceridemia ( ≥ 150 mg/dL)	Normal TG (%)/ High TG (%)	19 (67.9)/ 9 (32.1)	19 (67.9)/ 9 (32.1)	20 (58.8)/ 14 (41.2)	0.654
	Model 1 OR (95% CI)	Ref.	1.00 (0.326-3.07)	1.47 (0.519-4.20)	
	Model 2 OR (95% CI)	Ref.	0.808 (0.238-2.74)	1.26 (0.422-3.76)	
Hyperglycemia ( ≥ 100 mg/dL)	Normal FBS(%)/ High FBS (%)	12 (42.9)/ 16 (57.1)	12 (42.9)/ 16 (57.1)	20 (58.8)/ 14 (41.2)	0.270
	Model 1 OR (95% CI)	Ref.	1.00 (0.347-2.88)	0.525 (0.191-1.44)	
	Model 2 OR (95% CI)	Ref.	0.978 (0.311-3.07)	0.550 (0.188-1.60)	
Low HDL-C ( < 50 mg/dL)	Normal HDL(%)/ low HDL (%)	13 (46.4)/ 15 (53.6)	7 (25)/ 21 (75)	16 (47.1)/ 18 (52.9)	0.951
	Model 1 OR (95% CI)	Ref.	2.60 (0.838-8.07)	0.975 (0.358-2.65)	
	Model 2 OR (95% CI)	Ref.	3.16 (0.88-11.29)	0.996 (0.340-2.91)	
Central obesity (waist circumference > 88 cm)	Normal (%)/Obese (%)	1 (3.6)/ 27 (96.4)	0 (0)/ 28 (100)	0 (0)/ 34 (100)	0.990
	Model 1 OR (95% CI)	Ref.	-	-	
	Model 2 OR (95% CI)	Ref.	-	-	
HTN ( ≥ 130/85 mm Hg)	Normal (%)/ HTN (%)	25 (89.3)/ 3 (10.7)	21 (75)/ 7 (25)	24 (70.6)/ 10 (29.4)	0.142
	Model 1 OR (95% CI)	Ref.	2.77 (0.638-12.10)	3.47 (0.851-14.17)	
	Model 2 OR (95% CI)	Ref.	2.39 (0.460-12.48)	3.11 (0.691-14.07)	
MetS	Healthy (%)/MetS (%)	16 (57.1)/ 12 (42.9)	10 (35.7)/ 18 (64.3)	17 (50)/ 17 (50)	0.733
	Model 1 OR (95% CI)	Ref.	2.40 (0.818-7.03)	1.33 (0.488-3.64)	
	Model 2 OR (95% CI)	Ref.	2.69 (0.797-9.12)	1.22 (0.411-3.58)	

Abbreviations: HTN, hypertension; HDL-C, high density lipoprotein cholesterol; MetS, metabolic syndrome; BDNF, brain derived neurotrophic factor; BMI, body mass index Data were reported based on the binary logistic regression analysis. Model 1: Crude model Model 2: Adjusted for BMI, Age, physical activity level and energy intake

## Discussion

In the present study we investigated the association of serum neurotrophic factors and gastric hormones with CMR factors among ninety apparently healthy women. In the current study, risk of MetS was considerably lower among participants only in the second tertile of serum obestatin. However, the association between increased serum obestatin with decreased MetS risk were not statistically significant. Also, women who were in the second and third tertiles of serum obestatin levels had a significant lower risk for development of hypertriglyceridemia compared to the reference group. On the other hand, participants in the highest tertile of serum ghrelin had a significant lower risk of hypertriglyceridemia, hyperglycemia, low HDL-C, and MetS.

Obestatin and ghrelin are secreted from a same source and encoded by identical gene, but they have opposite physiological roles.^19^ Razzaghy-Azar et al showed that obestatin levels were considerably higher in obese compared to the normal children, while ghrelin levels were considerably lower in obese children compared to the controls.^20^ Ghrelin levels in obese children with MetS was lower than in obese children without MetS.^20^ Also, they suggested that the balance between ghrelin and obestatin seems to be an important factor in obesity and its associated complications.^20^ Mora et al showed that obestatin level was considerably higher in aged women with MetS, probably due to the role of this hormone in control of adiposity.^21^ On the other hand, one study found that decreased serum obestatin level was associated with atherosclerosis of the carotid artery, increased insulin resistance, and MetS in subjects with TRIB3 RR84 genotype polymorphism which indicates the role of genetics in the effects of obestatin on cardio-metabolic function.^22^ It has been suggested that obestatin could have protective effects on cardiomyocytes, possibly due to the its anti-apoptotic, anti-inflammatory, and anti-cytotoxic effects due to the increased Bcl-2 and Bax levels, expression of active caspase-3 as well as the increment of profibrogenic growth factors, such as transforming growth factor beta 1 (TGFβ1) and connective tissue growth factor (CTGF).^23,24^

Recent evidence has been suggested that ghrelin has critical role in glucose homeostasis and insulin secretion.^25^ Also, ghrelin can exert anti-apoptotic effects on endothelial cells via the PI3-kinase/Akt-pathway involved in insulin signaling.^26^ On the other hand, Agnew A.et al reported that treatment with obestatin analogues considerably improved plasma TG levels in rats.^27^ Also, Heshmatet al showed that the increase in plasma ghrelin level was associated with higher levels of HDL-C^28^ which confirm the findings of our study in this regard. However, plasma HDL-C is unlikely to be related to ghrelin levels. Recent studies have been shown that ghrelin can bind to HDL-C particles and increase its plasma concentration.^29^ So, it is possible that these HDL-C particles act as plasma carriers of ghrelin.^29^ On the other hand, based on in-vivo evidence, sustained administration of un-acylated ghrelin in high fat diet-treated mice, could enhance insulin signaling in skeletal muscle downstream of the mammalian target of rapamycin (mTORC) complexes and increase glucose uptake via insulin-simulated pathway.^30^ These findings are accompanied to the clinical evidence about the link of un-acylated ghrelin with whole-body insulin sensitivity.^31^ As there is a close link between insulin-resistance development and prevalence of hypertriglyceridemia and hyperglycemia^32^, this could be justified some part of our findings about the significant lower risk of hypertriglyceridemia, hyperglycemia, low HDL-C, and MetS in the highest tertile of serum ghrelin. However, we have not differentiated between acylated and un-acylated ghrelin in the present study and more detailed studies are needed to conclude the metabolic effect of ghrelin in this regard.

In the present study, there was no considerable association between serum obestatin tertiles and HTN risk. However, an increase in plasma ghrelin levels was associated with an improvement in BP, but these effects were not statistically considerable. Experimental evidence has shown that ghrelin has a beneficial effect on BP.^33^ There are too limited studies in this regard. Generally, there is an inverse association between high ghrelin levels and BP.^34,35^ Direct vasodilator effects, compact on renal diuresis, and modulation of the sympathetic nervous system are possible suggested mechanisms about the effect of ghrelin on lowering BP.^34^

In the present study, women who were in the higher tertile of serum NGF had a significant lower risk of hypertriglyceridemia and MetS compared to the reference group. Also, risk of hyperglycemia was considerably lower among the participants only in the second tertile of serum NGF compared to the reference group. On the other hand, there were no significant associations between the serum NGF tertiles and risk of HTN, central obesity, and low HDL-C.

Previous studies have been reported that NGF levels are associated with cardio-metabolic status.^36^ Animal studies have shown that lowering NGF levels leads to increased blood sugar and insulin resistance in diabetes.^37^ One study found that elevated plasma NGF levels in patients with MetS were considerably higher than in normal individuals.^10^ It has been suggested that NGF can be involved in cellular function by initiating phosphorylation of the protein cascade.^38^ NGF can also affect some metabolic conditions such as diabetes, obesity and MetS.^38^ Inconsistencies between findings of different studies is probably due to the different health/disease status in their participants.^9^

NGF has a role as a response protein in adipocyte inflammation.^9^ At the early stage of MetS, inflammation-induced hyper-neurothrophinemia is one the pathways to fight against inflammation.^9^ But, at the late stage of MetS, this response ultimately exhausts and leads to hyponeurothrophinemia. So, the protective role of NGF against inflammation and stress in MetS and obesity should be investigated in more details. On the other hand, NGF has structural homology with proinsulin.^38^ Also, pancreatic β cells synthesized and secreted NGF similar to insulin. NGF has role in fine -tuning insulin secretion.^38^ Experimental studies showed that NGF can promote glucose-simulated insulin secretion via receptor-mediated intracellular pathways.^38^ So, this can justify the regulatory role of NGF in insulin secretion and subsequent metabolism such as glucose and lipid metabolism. But finding the exact mechanism of this hypothesis needs more detailed studies.

In the present study we conducted the risk assessment analysis of BDNF, NGF, obestatin and ghrelin with CMR factors among apparently healthy women. There are so limited risk assessment studies, specially regression risk assessment, on the mentioned adipokines and neurotrophins and components of MetS. Despite intriguing results, the current findings need approving by large prospective studies and clinical trials. Conducting research only on females (to control confounding effect of gender) and recruitment of participants from a single dietary counselling center, are another limitations of the present study. Also, it’s necessary to consider different genotypes of mentioned hormonal and neurotrophic factors in future studies.

## Conclusion

In the current study, serum levels of obestatin, NGF, and ghrelin were associated with some CMR factors such as hypertriglyceridemia and MetS. It seems that these factors are associated with metabolic regulation. However, further studies are needed to substantiate this claim.

## Acknowledgements

This research was supported by Research Deputy of Tabriz University of Medical Sciences, Tabriz, Iran and Student Research Committee of Tabriz University of Medical Sciences, Tabriz, Iran.

## Funding

This research was supported by Research Deputy of Tabriz University of Medical Sciences, Tabriz, Iran and Student Research Committee of Tabriz University of Medical Sciences, Tabriz, Iran.

## Ethical approval

The Ethical Committee of Tabriz University of Medical Sciences, Tabriz, Iran approved the study protocol (Ethical code: TBZMED.REC.1394.111).

## Competing interest

None.

## References

[1] Wood LG (2015). Metabolic dysregulation. Driving the obese asthma phenotype in adolescents? Am J Respir Crit Care Med.

[2] Daneshpour MS, Fallah MS, Sedaghati-Khayat B, Guity K, Khalili D, Hedayati M (2017). Rationale and design of a genetic study on cardiometabolic risk factors: protocol for the Tehran Cardiometabolic Genetic Study (TCGS). JMIR Res Protoc.

[3] Palavra F, Reis F, Marado D, Sena A. Biomarkers of Cardiometabolic Risk, Inflammation and Disease. Springer; 2015.

[4] Bathina S, Das UN (2015). Brain-derived neurotrophic factor and its clinical implications. Arch Med Sci.

[5] Jabbari M, Kheirouri S, Alizadeh M (2018). Decreased serum levels of ghrelin and brain-derived neurotrophic factor in premenopausal women with metabolic syndrome. Lab Med.

[6] Steckiewicz KP, Barcińska E, Woźniak M (2018). Nerve growth factor as an important possible component of novel therapy for cancer, diabetes and cardiovascular diseases. Cell Mol Biol (Noisy-le-grand).

[7] Rozanska O, Uruska A, Zozulinska-Ziolkiewicz D (2020). Brain-derived neurotrophic factor and diabetes. Int J Mol Sci.

[8] László A, Lénárt L, Illésy L, Fekete A, Nemcsik J (2019). The role of neurotrophins in psychopathology and cardiovascular diseases: psychosomatic connections. J Neural Transm (Vienna).

[9] Hristova MG (2013). Metabolic syndrome--from the neurotrophic hypothesis to a theory. Med Hypotheses.

[10] Atanassova P, Hrischev P, Orbetzova M, Nikolov P, Nikolova J, Georgieva E (2014). Expression of leptin, NGF and adiponectin in metabolic syndrome. Folia Biol (Krakow).

[11] Kheirouri S, Jabbari M, Alizadeh M (2018). Obestatin and nerve growth factor in patients with metabolic syndrome. Prog Nutr.

[12] Yu AP, Ugwu FN, Tam BT, Lee PH, Ma V, Pang S (2020). Obestatin and growth hormone reveal the interaction of central obesity and other cardiometabolic risk factors of metabolic syndrome. Sci Rep.

[13] Stasi C, Milani S (2016). Functions of ghrelin in brain, gut and liver. CNS Neurol Disord Drug Targets.

[14] El Sawy SA, El-Sherbiny RA, El-Saka MH, El-Shaer RA (2016). Effect of obestatin on normal, diabetic, and obese male albino rats. Tanta Med J.

[15] Mihalache L, Gherasim A, Niţă O, Ungureanu MC, Pădureanu SS, Gavril RS (2016). Effects of ghrelin in energy balance and body weight homeostasis. Hormones (Athens).

[16] da Silva Pereira JA, da Silva FC, de Moraes-Vieira PMM (2017). The impact of ghrelin in metabolic diseases: an immune perspective. J Diabetes Res.

[17] Tabatabaei-Malazy O, Saeedi Moghaddam S, Rezaei N, Sheidaei A, Hajipour MJ, Mahmoudi N (2021). A nationwide study of metabolic syndrome prevalence in Iran; a comparative analysis of six definitions. PLoS One.

[18] Vasheghani-Farahani A, Tahmasbi M, Asheri H, Ashraf H, Nedjat S, Kordi R (2011). The Persian, last 7-day, long form of the International Physical Activity Questionnaire: translation and validation study. Asian J Sports Med.

[19] Farajallah A, Shanableh S (2017). Ghrelin structure and it’s receptors: a review. J Res Pharm Sci.

[20] Razzaghy-Azar M, Nourbakhsh M, Pourmoteabed A, Nourbakhsh M, Ilbeigi D, Khosravi M (2016). An evaluation of acylated ghrelin and obestatin levels in childhood obesity and their association with insulin resistance, metabolic syndrome, and oxidative stress. J Clin Med.

[21] Mora M, Granada ML, Roca M, Palomera E, Puig R, Serra-Prat M (2013). Obestatin does not modify weight and nutritional behaviour but is associated with metabolic syndrome in old women. Clin Endocrinol (Oxf).

[22] Cui AD, Gai NN, Zhang XH, Jia KZ, Yang YL, Song ZJ (2012). Decreased serum obestatin consequent upon TRIB3 Q84R polymorphism exacerbates carotid atherosclerosis in subjects with metabolic syndrome. Diabetol Metab Syndr.

[23] Zhang Q, Dong XW, Xia JY, Xu KY, Xu ZR (2017). Obestatin plays beneficial role in cardiomyocyte injury induced by ischemia-reperfusion in vivo and in vitro. Med Sci Monit.

[24] Aragno M, Mastrocola R, Ghé C, Arnoletti E, Bassino E, Alloatti G (2012). Obestatin induced recovery of myocardial dysfunction in type 1 diabetic rats: underlying mechanisms. Cardiovasc Diabetol.

[25] Alamri BN, Shin K, Chappe V, Anini Y (2016). The role of ghrelin in the regulation of glucose homeostasis. Horm Mol Biol Clin Investig.

[26] Kurowska P, Mlyczynska E, Rak A (2019). Effect of ghrelin on the apoptosis of various cells A critical review. J Physiol Pharmacol.

[27] Agnew A, Calderwood D, Chevallier OP, Greer B, Grieve DJ, Green BD (2011). Chronic treatment with a stable obestatin analog significantly alters plasma triglyceride levels but fails to influence food intake; fluid intake; body weight; or body composition in rats. Peptides.

[28] Heshmat R, Shafiee G, Qorbani M, Azizi-Soleiman F, Djalalinia S, Esmaeil Motlagh M (2016). Association of ghrelin with cardiometabolic risk factors in Iranian adolescents: the CASPIAN-III study. J Cardiovasc Thorac Res.

[29] Yin Y, Zhang W (2016). The role of ghrelin in senescence: a mini-review. Gerontology.

[30] Gortan Cappellari G, Zanetti M, Semolic A, Vinci P, Ruozi G, Falcione A (2016). Unacylated ghrelin reduces skeletal muscle reactive oxygen species generation and inflammation and prevents high-fat diet-induced hyperglycemia and whole-body insulin resistance in rodents. Diabetes.

[31] Barazzoni R, Gortan Cappellari G, Semolic A, Ius M, Mamolo L, Dore F (2016). Plasma total and unacylated ghrelin predict 5-year changes in insulin resistance. Clin Nutr.

[32] Ormazabal V, Nair S, Elfeky O, Aguayo C, Salomon C, Zuñiga FA (2018). Association between insulin resistance and the development of cardiovascular disease. Cardiovasc Diabetol.

[33] Wang Q, Sui X, Chen R, Ma PY, Teng YL, Ding T (2018). Ghrelin ameliorates angiotensin II-induced myocardial fibrosis by upregulating peroxisome proliferator-activated receptor gamma in young male rats. Biomed Res Int.

[34] Mao Y, Tokudome T, Kishimoto I (2016). Ghrelin and blood pressure regulation. Curr Hypertens Rep.

[35] Kolahdouz-Mohammadi R, Malekahmadi M, Clayton ZS, Sadat SZ, Pahlavani N, Sikaroudi MK (2020). Effect of egg consumption on blood pressure: a systematic review and meta-analysis of randomized clinical trials. Curr Hypertens Rep.

[36] Frohlich J, Chaldakov GN, Vinciguerra M (2021). Cardio-and neurometabolic adipobiology: consequences and implications for therapy. Int J Mol Sci.

[37] Bell B, Ybarbo K, Lee J, Huo Y, Graham J, Stanhope K (2021). Circulating NGF is correlated with indexes of diabetes progression and P2X3 expression in UCD-T2DM rats. FASEB J.

[38] Ding XW, Li R, Geetha T, Tao YX, Babu JR (2020). Nerve growth factor in metabolic complications and Alzheimer’s disease: physiology and therapeutic potential. Biochim Biophys Acta Mol Basis Dis.

